# Potential Neuroprotective Effect of the Endocannabinoid System on Parkinson's Disease

**DOI:** 10.1155/2024/5519396

**Published:** 2024-07-25

**Authors:** María Fernanda Urmeneta-Ortíz, Aldo Rafael Tejeda-Martínez, Orfil González-Reynoso, Mario Eduardo Flores-Soto

**Affiliations:** ^1^ Chemical Engineering Department, University Center for Exact and Engineering Sciences University of Guadalajara, Blvd. M. García Barragán # 1451, Guadalajara C.P. 44430, Jalisco, Mexico; ^2^ Cellular and Molecular Neurobiology Laboratory Neurosciences Division Western Biomedical Research Center (CIBO) Mexican Social Security Institute, Sierra Mojada #800, Independencia Oriente, Guadalajara 44340, Jalisco, Mexico

## Abstract

Parkinson's disease (PD) is a neurodegenerative disorder characterized by alterations in motor capacity resulting from a decrease in the neurotransmitter dopamine due to the selective death of dopaminergic neurons of the nigrostriatal pathway. Unfortunately, conventional pharmacological treatments fail to halt disease progression; therefore, new therapeutic strategies are needed, and currently, some are being investigated. The endocannabinoid system (ECS), highly expressed in the basal ganglia (BG) circuit, undergoes alterations in response to dopaminergic depletion, potentially contributing to motor symptoms and the etiopathogenesis of PD. Substantial evidence supports the neuroprotective role of the ECS through various mechanisms, including anti-inflammatory, antioxidative, and antiapoptotic effects. Therefore, the ECS emerges as a promising target for PD treatment. This review provides a comprehensive summary of current clinical and preclinical evidence concerning ECS alterations in PD, along with potential pharmacological targets that may exert the protection of dopaminergic neurons.

## 1. Introduction

Parkinson's disease (PD) is a neurodegenerative disorder predominantly affecting the elderly population. This pathology is mainly characterized by motor symptoms such as rigidity, bradykinesia, tremor at rest, and postural deterioration. Additional symptoms often include loss of balance, hypotension, hyposmia, dysphagia, cognitive impairment, and affective disorders, among others. The main pathophysiological features of PD are the cytoplasmic accumulation of the *α*-synuclein protein, known as Lewy bodies, and the progressive loss of dopaminergic neurons in the substantia nigra *pars compacta* (SN*pc*), resulting in diminished dopamine levels. These changes affect various brain structures, including the cerebellum, the thalamus, the hypothalamus, parts of the cerebral cortex, the spinal cord, the locus coeruleus, and basal ganglia (BG) [1, 2].

Numerous interconnected mechanisms contribute to the degeneration of nigrostriatal dopaminergic neurons, acting simultaneously. These include mitochondrial dysfunction, oxidative stress, proteostasis alteration, inflammation, microglial dysregulation, and excitotoxicity. The study of these mechanisms has identified molecular targets and neurotransmission systems influencing the genesis and progression of PD. This understanding shapes the development of future neuroprotective strategies based on multifactorial pharmacology [3].

The current pharmacological treatments for PD are limited. Levodopa (L-DOPA), the gold standard for advanced-stage symptomatic relief, does not impede disease progression. Long-term L-DOPA use leads to levodopa-induced dyskinesias (LIDs), prompting physicians to initially prescribe less effective dopamine agonists or monoamine oxidase inhibitors, which also fail to halt disease progression and ultimately lead to L-DOPA administration and LID [4]. Consequently, research is ongoing for new therapeutic alternatives to stop or delay PD development while protecting dopaminergic nerve cells to support proper motor function. In this review, we summarize clinical and preclinical findings on the involvement of the ECS through its endogenous ligands, catabolic enzymes, and receptors as potential therapeutics for PD [3, 5].

## 2. Endocannabinoid System

ECS is a molecular signaling system present in a diverse array of human cells and tissues. It is also present in many other mammals, and its presence in rodents is very suitable due to its wide use in preclinical research. The ECS plays a pivotal role in modulating various physiological functions, including mood, pain, feeding behavior, and motor control, while also contributing to the pathogenesis of several psychiatric and neurological disorders [6]. The ECS is a complex regulatory network comprising endogenous ligands known as endocannabinoids (eCBs), receptors, and diverse proteins responsible for the eCB synthesis, transport, and degradation [3].

### 2.1. Ligands

Endogenous cannabinoids are lipophilic molecules produced tonically and on demand from lipid precursors in the membrane of postsynaptic neurons and glial cells, responding to physiological and pathological stimuli. In the nervous system, eCBs are recognized for their ability to modulate neural activity, acting also as regulators of the immune response and antioxidant agents, endowing them with neuroprotective properties. These actions depend on their interaction with receptors and are tightly regulated by reuptake (mediated by selective transporters in neuronal and glial cells) and subsequent inactivation through enzymatic degradation [7]. The most abundant and well-described eCBs in the brain parenchyma are 2-arachidonoylglycerol (2-AG) and anandamide (AEA). Together with other ECS components, they significantly regulate motor function within the BG circuit [8].

### 2.2. Receptors

Two proteins, cannabinoid receptors type 1 (CB1) and 2 (CB2), originating from different genes, have been characterized and confirmed by using crystallography. Both receptors possess seven transmembrane domains and are coupled to G proteins, resulting in predominantly modulatory effects that vary depending on the cell lineage and the type of G protein coupled [9, 10]. These receptors are expressed on the cell membrane of numerous tissues throughout the organism [11, 12]. While other receptors have been described, only CB1 and CB2 are considered canonical and not orphan [12, 13]. Notably, eCB can interact with non-CB1/non-CB2 targets, including transient receptor potential (TRP) channels [14], peroxisome proliferation activated receptor (PPAR)-*α* and PPAR-*γ* [15], as well as the G protein-coupled receptor (GPR) 18, GPR55, and GPR119 [7].

### 2.3. Biotransformation Enzymes

Two additional components of the ECS are biosynthesis and degradation enzymes. Unlike classical neurotransmitters and neuropeptides, eCBs are not stored in vesicles within axon terminals; instead, they are synthesized on demand in cell bodies and dendrites. They are subsequently released and exert immediate action as signaling molecules. Endocannabinoids are endogenous signaling lipid molecules generated from phospholipid precursors in the cell membrane, primarily derived from arachidonic acid conjugated with ethanolamine, glycerol, other fatty acids, and even neurotransmitters [16, 17].

The principal eCBs include anandamide (AEA, arachidonyl ethanolamine) and 2-arachidonylglycerol (2-AG). The eCB family also encompasses virodhamine, noladineter, N-arachidonoyl dopamine (NADA), homo-*γ*-linolenylethanolamide (HEA), docosatetraenoylethanolamide (DEA), and related compounds such as palmitoylethanolamide (PEA) and oleoylethanolamide (OEA) [11, 18, 19]. The synthesis of 2-AG primarily involves activating the phospholipase C*β*/diacylglycerol lipase (PLC*β*/DGL) pathway. The DGL enzyme coexists in two isoforms, DGL*α* and DGL*β*, with DGL*α* being the dominant isoform in producing 2-AG within the brain parenchyma in most regions. However, recent work carried out by Liu et al. has demonstrated that DGL*β* is particularly relevant in the SN*pc*, substantia nigra *pars reticulata* (SN*pr*), and striatum, uncovering changes in the gene coding for this isoform in some hereditary cases of PD. This discovery led to the generation of a new PD model through conditional knockout of this gene, specifically in the SN [20]. In contrast, AEA is primarily synthesized via N-acyl phosphatidylethanolamine phospholipase D (NAPE-PLD) in conjunction with other N-acylethanolamides such as oleylethanolamide (OEA) and palmitoylethanolamide (PEA). Regarding eCB catabolism, monoacylglycerol lipase (MAGL) is the predominant enzyme degrading 2-AG to arachidonic acid (AA) and glycerol. In contrast, fatty acid amide hydrolase (FAAH) largely catalyzes the degradation of AEA, OEA, and PEA to ethanolamine and the corresponding fatty acid (AA, oleic acid, and palmitic acid, respectively) [7].

### 2.4. Transport Mechanism

**Figure 1:**
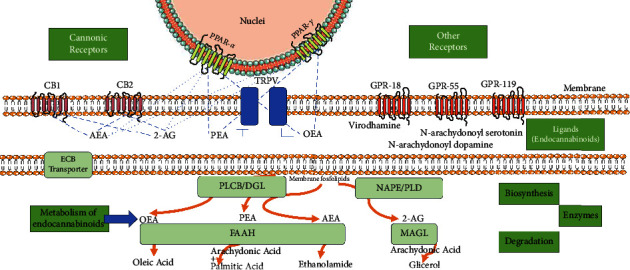
Main components of the endocannabinoid system. Main and most studied ECS components. Ligands (center of the figure) activate both canonical and noncanonical receptors (orphan GPR and nuclear PPAR receptors) and differentially modulate TRPV receptor activation. The biosynthesis and degradation of these ligands rely on diverse enzymes that degrade membrane phospholipids and generate precursors for other important lipidic mediators, such as arachidonic acid.

MAGL, FAAH, and other enzymes responsible for the degradation of eCB are localized within the intracellular compartment, suggesting the existence of cannabinoid transporters. While crystallography has not yet demonstrated the presence of a transporter, evidence supporting its existence arises from the discovery of a transcription variant of the FAAH gene that retains its complete structure except for the catalytic site, coupled with the activity of specific molecules that can only be explained by the involvement of transporter proteins [21]. A brief scheme of the main components of the ECS centered on ligands and their targets is presented in Figure 1.

Current evidence, recapitulated from studies on patients with PD and animal models of parkinsonism, indicates that ECS undergoes modifications within the BG circuit. These alterations have been investigated regarding changes in receptor expression and the activity of biosynthesis and degradation enzymes, leading to variations in ligand levels when comparing healthy and affected individuals. Some of these cases likely represent compensatory mechanisms following dopamine depletion, such as the case of AEA in the striatum. Others are more likely related to the manifestation of motor symptoms. These observations provide a rationale for various methodological approaches to enhance ECS signaling, which have demonstrated positive antiparkinsonian effects [5]. Consequently, the ECS is considered a significant pharmacological target to alleviate motor symptoms and inhibit neurodegeneration progression. It is noteworthy that the distribution of cannabinoid receptors, their neuromodulatory mechanisms, including anti-inflammatory and antioxidant properties, and their role in the pathophysiology of PD have been the primary focus of cannabinoid system research, as will be described later.

## 3. Alterations of the Endocannabinoid System in Parkinson's Disease

It is well established that eCBs are highly expressed in the BG, and their levels are altered in individuals with PD and animal models of parkinsonism induction. Consistent with this, a clinical study showed that in cerebrospinal fluid (CSF) samples from PD patients, AEA levels were twice as high as in controls, with no correlation between this increase and disease progression. Furthermore, these changes did not manifest in patients undergoing pharmacological treatment with L-DOPA or dopamine agonists [22]. Additionally, studies conducted by Marchioni et al. in 2020 demonstrated reduced levels of 2-AG and increased levels of AEA in CSF of PD patients under L-DOPA treatment without LID, compared to healthy controls. Notably, these alterations were not modified in patients with LID [23]. Also, preclinical studies in animal models of parkinsonism induction have exhibited alterations in the levels of these eCBs in the BG circuit in a region-dependent fashion.

In the striatum of monkeys treated with 1-methyl-4-phenyl-1,2,3,6-tetrahydropyridine (MPTP), a neurotoxin causing dopaminergic cell death, both AEA and 2-AG levels were increased and reverted to basal levels after levodopa treatment, without correlation with the development of LID [24]. Di Marzo et al. in 2000 demonstrated an increase in 2-AG levels in the globus pallidus of rats treated with reserpine, suggesting that an increase in 2-AG levels might be a consequence of more significant activity in the BG indirect pathway [8]. Moreover, in the striatum of 6-hydroxydopamine (6-OHDA)-lesioned rats, elevated AEA levels were found due to reduced activity of the anandamide membrane transporter and the degradation enzyme FAAH. This led to an overactivation of spontaneous glutamatergic activity [25]. These changes were reversed entirely after chronic L-DOPA treatment, suggesting that the increased AEA levels imply a compensatory mechanism for abnormal glutamatergic input [26].

In a series of studies conducted by Concannon et al. employing various methodologies for parkinsonism induction, alterations in eCB levels in the striatum differed amongst models. AEA levels increased in the striatum of rats lesioned intrastrially with rotenone and lipopolysaccharide (LPS), but this effect was not observed in animals receiving 6-OHDA. Additionally, 2-AG levels were elevated only in animals treated with LPS, while the content of PEA and OEA was increased in all models, albeit in different concentrations. Therefore, special care should be taken when interpreting the results [27, 28].

A recently published study by Liu et al. demonstrated that, in SN*pc* neurons, the DGL*β* isoform predominates in 2-AG synthesis and that the loss of function of this enzyme, induced by genetic mutations, is associated with familial forms of early-onset autosomal recessive parkinsonism. In the same study, the authors showed that pharmacological inhibition of the enzyme MAGL improves the motor response of rodents with a specific deletion of the *Dglβ* gene in dopaminergic neurons of the SN*pc* by enhancing the activity of dopaminergic neurons and the consequent release of dopamine [20]. Additionally, Navarrete et al. observed a decrease in MAGL in the SN*pc* and an increase in striatum samples of PD patients [29].

Regarding cannabinoid receptors, *in vivo* studies in patients with PD revealed decreased CB1 receptors in the SN*pc*, not correlated with disease duration [30]. In animal studies, Walsh et al. explored the temporal and regional patterns of CB1 receptor expression in rats unilaterally lesioned with 6-OHDA. CB1 levels did not change in the striatum but were diminished in internal globus pallidus and SN*pr*. In contrast, in external globus pallidus, the expression levels were increased, although not sustained over time [31]. In *postmortem* studies, CB2 receptors showed an increased expression in astrocytes of the SN*pc* [29].

Several experimental pieces of evidence have demonstrated the pivotal involvement of astrocytes and microglia cells in the genesis of PD. During the early stages of the disease, research has shown that astrocytes absorb altered *α*-synuclein from axon terminals [32], leading to neurodegenerative processes through the production of proinflammatory cytokines [33] and microglial activation [34]. Activated microglia further contribute significantly to the loss of dopaminergic neurons of the SN*pc* and dopaminergic denervation in the striatum [35, 36]. Moreover, various preclinical and clinical evidence has demonstrated the presence of the CB2 receptor in microglia cells and astrocytes [37], suggesting a neuromodulatory role in both pro- and anti-inflammatory events in PD. Furthermore, Price et al. demonstrated an increase in CB2 receptor expression in microglia cells after administering MPTP. Agonism of the CB2 receptor with JWH015 (4 mg/kg, intraperitoneal) reduced MPTP-induced microglial activation, while genetic ablation of CB2 receptors exacerbated MPTP systemic toxicity. A recent study also reveals a significant increase in gene transcription and CB2 protein expression after intrastriatal injection with 6-OHDA or bacterial LPS. This increase is accompanied by enhanced microglial expression and activation in the SN*pc* and the striatum, suggesting that the CB2 receptor is a potential pharmacological target to inhibit both inflammatory and oxidative stress events [27].

Similarly, Concannon et al. employing different models of parkinsonism induction reported an upregulation of CB2 receptors in rats' SNpc. This response was slightly more pronounced when an inflammatory stimulus such as LPS was used compared to 6-OHDA and rotenone [28].

## 4. Evidence of the Neuroprotective Effect of the Endocannabinoid System on Parkinson's Disease

### 4.1. Endocannabinoids

The neuroprotectant effect of eCBs has been thoroughly examined by various authors. These studies have revealed that eCBs exert anti-inflammatory, antioxidative, and antiapoptotic effects while inhibiting mitochondrial dysfunction and excitotoxicity-induced death, mechanisms implicated in the pathophysiology of PD [3].

Regarding the neuroprotective capacity of 2-AG, a study conducted by Mounsey et al. in 2015 observed that treatment with MPTP (30 mg/kg, i.p. ×5 days) increases the levels of 2-AG in the midbrain of C57BL6/J mice from the second day of administration, maintaining elevated levels until the end of the treatment. This increase in 2-AG, as suggested by Mounsey et al., might be a compensatory mechanism for counteracting the oxidative damage induced by MPTP to prevent further harm. In line with this, the authors demonstrated that pretreatment with 2-AG (3 and 5 mg/kg, i.p.) prevented the death of dopaminergic neurons in the SN*pc* of MPTP-treated mice. This protective effect was shown to be potentiated by inhibiting the degradation of this eCB [38]. Furthermore, studies have indicated that direct manipulation of 2-AG levels by inhibiting their degradation not only results in symptomatic relief [20, 39–41] but also exerts a neuroprotective effect [38, 42–44].

Preclinical studies demonstrated that treatment with JZL184, a MAGL inhibitor, increases 2-AG levels and prevents the death of neuronal cells of the nigrostriatal pathway at doses between 8 and 40 mg/kg [38, 43]. However, high doses or prolonged use of JZL184 can inhibit the enzyme FAAH [45]. While this represents an additional pathway for 2-AG degradation, FAAH exhibits a higher affinity for AEA and other fatty acid ethanolamines. Consequently, inhibiting FAAH catalytic activity may lead to an increase in the levels of these eCBs [43]. Notably, Fernández-Suárez et al. demonstrated that FAAH inhibition does not prevent the toxicity induced by MPTP plus probenecid (MPTPp), aligning with results from other research groups [40, 41]. Treatment with JZL184 increases 2-AG and other eCB levels, making it difficult to determine if the neuroprotective effect should be attributed solely to 2-AG. In 2017, Pasquarelli et al. evaluated the impact of MAGL inactivation with the inhibitor KML29 (10 mg/kg, i.p.) in C57BL/6J mice undergoing chronic treatment with MPTPp (10 mg/kg and 250 mg/kg, i.p., respectively). The results demonstrated an improvement in motor response and a reduction in dopamine depletion in the striatum. However, it is noteworthy that the authors did not assess the extent of dopaminergic neuronal death in the SN*pc* [41].

The mechanisms underlying the neuroprotective role of 2-AG against dopaminergic neuron death have received limited attention. In a study conducted by Aymerich et al. in 2016, inhibition of MAGL with JZL184 (0.1 and 1 *μ*M) was shown to counteract the toxicity induced by 1-methyl-4-phenylpyridinium (MPP^+^: the putative toxic metabolite of the neurotoxin MPTP) in the SH-SY5Y cell line. Notably, this effect was found to be dependent on CB2 receptor agonism. The study revealed that JZL184 increased mRNA levels of CB2 receptors, and the antagonism induced by the addition of AM630 (1 *μ*M) completely inhibited the neuroprotective effect of JZL184 (0.1 *μ*M) [46]. Data collected by Nomura et al. in 2011 demonstrated that both genetic (Mgll −/− mice) and pharmacological (pretreatment with JZL184, 40 mg/kg, v.o.) inactivation of MAGL in an acute MPTP-induced parkinsonism model (15 mg/kg, i.p.) led to reducing levels of AA, prostaglandins, and proinflammatory cytokines. This suggests that blocking 2-AG degradation contributes to decreased inflammatory response triggered by dopaminergic neuron death [42]. Consistent with this, Mounsey et al. demonstrated that inactivation of the cyclooxygenase-2 enzyme (an enzyme involved in the conversion of AA to prostaglandins) with a selective inhibitor of inflammatory mediator cyclooxygenase-2: DFU (25 mg/kg, i.p.) potentiated the neuroprotective effect of MAGL inhibition [38].

Furthermore, the Fernández-Suárez research group observed increased immunoreactivity of microglia and astrocytes in the SN*pc* and striatum, along with elevated expression levels of transforming growth factor-*β* (TGF-*β*) and glial cell line-derived neurotrophic factor (GDNF) in the striatum of mice with induced parkinsonism [43]. These findings suggest that the neuroprotective and antiparkinsonian effects achieved through MAGL inhibition are associated with an anti-inflammatory response mediated by increased 2-AG levels and its interaction with CB2 receptors.

The current evidence on the neuroprotective effect of AEA in animal models of parkinsonism is limited and primarily based on observations following the inhibition of its enzymatic degradation. Furthermore, the mechanisms underlying the protective activity of the nigrostriatal pathway remain unclear. Escamilla-Ramirez et al. employed a potent irreversible FAAH inhibitor, URB597, to increase AEA tone in C56BL/6NHsd mice with acute MPTP-induced parkinsonism (40 mg/kg, s.c.). Results showed that treatment with URB597 (0.3 mg/kg, i.p., ×5 days), before or after neurotoxin administration, improved motor performance and reduced oxidative damage by preventing impairment of the nigrostriatal pathway [44]. Similarly, Viveros-Paredes et al. reported that 30-day pretreatment with URB597 (0.2 mg/kg, i.p., administered every other day) counteracted nigrostriatal pathway denervation, damage-associated immune response, and motor deficit by preventing neuronal death induced by MPTP (30 mg/kg, i.p., ×5 days) [47]. Conversely, Celorrio et al. found that chronic administration of URB597 (1 mg/kg, i.p., five times/week during 5-week MPTPp treatment) did not prevent dopaminergic degeneration. However, AEA degradation blockade improved motor function in mice with hemiparkinsonism [40].

FAAH is also implicated in the degradation of other members of the fatty acid ethanolamide family. URB597-induced FAAH inactivation significantly elevated levels of AEA, OEA, and PEA in the midbrain of mice with parkinsonism [40]. OEA has been described as an agonist at PPAR-*α* and differs from AEA by functioning as an antagonist of transient receptor potential vanilloid 1 (TRPV1) and lacking interaction with CB1 or CB2 receptors [48]. Gonzalez-Aparicio et al. investigated the protective effect of OEA at different concentrations (0, 5, 1 y 5 mg/kg) in rats lesioned with 6-OHDA (5 *μ*g/*μ*l intrastriatal). Their results demonstrated that intraperitoneal injection of OEA could penetrate the blood-brain barrier, reach active concentrations in the BG, and elicit anti-inflammatory/antioxidant responses. This resulted from the activation of PPAR-*α* receptors expressed by dopaminergic neurons in both the SN and the dopaminergic fibers in the dorsal striatum [49–52]. PPAR-*α* receptor activation decreases neuronal death through vascular protection and exerts vascular effects by reducing oxidative stress and preventing adhesion protein expression [53]. PPAR-*α* also regulates apoptosis and inflammation, inhibiting the transcription of inflammatory response genes by antagonizing the AP-1 and nuclear factor *κ*B signaling pathways (NF*κ*B) [54–56].

The investigations into PEA's neuroprotective and antiparkinsonian effects have mainly focused on its anti-inflammatory capacity, involving the activation of PPAR-*α* receptors. Esposito et al. evaluated the systemic administration of PEA (10 mg/kg, i.p., ×7 days starting 24 h postinsult with MPTP (80 mg/kg, i.p.)) in parkinsonian mice with *PPAR-α* gene deletion. Mice presented slightly more significant damage than induced by MPTP, and PEA treatment lost its beneficial effect, indicating that signaling mediated by PPAR-*α* activation is involved in neuroprotection mechanisms. In the same study, authors observed that PEA prevented apoptosis by modulating the Bax/Bcl-2 pathways, mechanisms largely dependent on the activation of PPAR-*α* [57]. Similarly, Cupri et al. assessed a micronized formulation of PEA (mPEA, 10 mg/kg, p.o. ×60 days) in aged CD1 mice (21-month-old). Old mice presented more significant cellular and behavioral impairment due to MPTP toxicity than young mice (3 months). mPEA pretreatment demonstrated a decrease in apoptotic death of SN*pc* neurons, an improvement in motor skills, a reduction in *α*-synuclein expression, and modulation of proinflammatory (TNF-*α* and IL-1*β*) and anti-inflammatory (IL-10) cytokines [57, 58]. Furthermore, the protective and antiparkinsonian effect of PEA described in the MPTP model was corroborated in 6-OHDA partially injured mice (4 *μ*g). Results showed reductions in oxidative stress, inflammation, endoplasmic reticulum stress, and apoptosis at the three doses (3, 10, and 30 mg/kg, s.c., ×8 days). The involvement of PPAR-*α* receptors was evidenced *in vivo* with GW7647 administration, a synthetic agonist (5 mg/kg, s.c.) exerting similar effects in every parameter, and *in vitro* in the SH-SY5Y cell line in which the addition of GW6471, a PPAR-*α* antagonist (0.3 and 1 *μ*M), prevented the protective effect of PEA (1 *μ*M) [59].

PEA degradation is also catalyzed by the enzyme N-acylethanolamine acid amidase (NAAA). In a recent study, researchers found an increase in NAAA levels in the cerebral cortex of *postmortem* patients with PD and exosomes derived from the blood of individuals in stages I and II of this disease [60]. Consistent with these findings, *in vitro* experiments demonstrated increased NAAA expression and decreased PEA concentration in SH-SY5Y cells stimulated with 6-OHDA (100 *μ*M). Interestingly, the addition of MPP^+^ (2 mM) stimulated enzyme expression but did not lead to a significant decrease in PEA content. *In vivo*, intrastriatal injection of 6-OHDA (6.4 *μ*g) increased NAAA immunoreactivity in dopaminergic neurons of the SN*pc* within the initial 48 hours and, two weeks later, in microglial cells confined to the SN*pc* and striatum.

Moreover, genetic and pharmacological inactivation of NAAA (twice daily administrations of ARN19702 at a dose of 30 mg/kg, i.p.) produced a neuroprotective and antiparkinsonian effect in the lesion model with 6-OHDA and toxicity induced by MPTP (72 mg/kg, i.p.). This study also demonstrated the involvement of PPAR-*α* receptors in the protective action of PEA. Specifically, the activation of these receptors promoted the survival of dopaminergic neurons, at least partially, by preserving their bioenergetic potential through normalizing the expression of the transcription coactivators PPAR coactivator (PGC)-1*α* and PGC-1*β*, which are suppressed by 6-OHDA. The combined evidence from Palese et al. coupled with previously described findings on the protective effect of PEA positions the NAAA-PEA-PPAR-*α* pathway as a promising therapeutic target for managing PD [60].

### 4.2. CB2

Under physiological conditions, CB2 receptors are expressed at low concentrations in microglia, astrocytes, and neurons of the hippocampus, striatum, brainstem, ventral tegmental area, and SN*pc*. However, under pathological conditions, such as PD, their expression predominantly increases in the glial cell lineage [28, 61]. This upregulation is interpreted as an endogenous protective response aimed at delaying or halting the progression of the disease by controlling the inflammatory response. Therefore, pharmacological manipulation of CB2 receptors represents a promising strategy for PD [62]. Several preclinical studies have reported that natural and synthetic agonists for CB2 receptors protect dopaminergic neurons and prevent motor dysfunction, possibly by reducing damage-related glial alterations [62–64]. These findings are supported by evidence that genetic ablation of the CB2 receptor exacerbates the toxicity of MPTP [63].

Regarding the mechanism by which CB2 agonism exerts neuroprotection, a recent study by Wang's group investigated the role of nuclear factor erythroid 2-related factor 2 (Nrf2) in the immunomodulatory effect induced by JWH133, a CB2 agonist, in a microglia culture treated with MPP^+^. The results indicated that the PI3K/Akt/Nrf2 pathway is involved in the polarization of microglia toward an anti-inflammatory phenotype [65]. Additionally, Yu et al. reported that the neuroprotective effect of GW842166x, another CB2 agonist, in the rodent MPTP model is associated with the capacity of CB2 receptors localized in dopaminergic neurons to reduce the action potential firing and the associated calcium overload that may cause cytotoxicity [64]. The main components of ECS and their alteration in patients with PD and some animal models for the pathology are presented in Table 1.

### 4.3. PPAR

PPAR is a nuclear receptor group that functions as transcription factors and requires the transcription coactivator PGC-1*α*. Their activation entails physiological activities primarily mediated by binding to peroxisome proliferation response element sites [67]. These receptors are divided into three subtypes: *α*, *β*/*δ*, and *γ*; their predominant cellular distribution in brain parenchyma is neuronal. Under physiological conditions, all three isotypes are present in astrocytes, but only isoform *α* is expressed in microglial cells in both human and mouse brains. However, in response to LPS, PPAR-*γ* increases in these cells region-dependent, while PPAR-*α* levels are maintained [68].

Various specific agonists for these receptors have been developed and utilized, especially for PPAR-*γ*. One of the most important is pioglitazone which has been associated with protective effects against PD in patients and has been already used for other diseases, suggesting that sustained receptor activation could be neuroprotective [69]. Pioglitazone has been effective in the treatment of neurodegenerative disorders at a preclinical level in nonhuman primates [70] and parkinsonism models [69]. Mechanisms underlying the antiparkinsonian effects of this activation have been explored. On one hand, pioglitazone has been proven to regulate neural plasticity and exhibit neuroprotective effects through anti-inflammatory and antioxidative mechanisms [69]. Regarding work in nonhuman primates, transient regulation of paraoxonase-2 expression has been described as a possible mechanism of neuroprotection due to PPAR-*γ* activation. This protein, widely expressed in regions with a high dopamine concentration, enhances coenzyme Q function at the mitochondrial level, potentially reducing oxidative stress [70]. On the other hand, molecules related to cannabinoids with activity solely on PPAR-*γ* receptors, such as cannabigerol quinone derivatives VCE-003.2 and VCE-004.8, have been produced [71, 72]. These compounds have exhibited anti-inflammatory effects along with neuroprotective effects and improvement of locomotor activity in parkinsonism models [15, 71–73]. In all these studies, these effects were explained by PPAR-*γ* activation; nevertheless, an antagonist of these receptors failed to fully deplete its activity, suggesting binding of the derivate in noncanonical regions [73].

### 4.4. TRPV1

TRPV1 is a nonselective cation channel activated by various endogenous and exogenous stimuli, including heat, natural vanilloids (capsaicin and resiniferatoxin), and eCB, such as AEA [74]. This receptor is widely expressed in sensory neurons, dopaminergic cells, astrocytes, and microglia within the nigrostriatal pathway. Its physiological role involves the modulation of pain perception, motor control, and inflammation [14]. Preclinical studies have shown that agonism of the TRPV1 receptor with capsaicin, a natural agonist, prevents the degeneration of dopaminergic cells in models of parkinsonism induction by inhibiting the inflammatory response. Specifically, it modulates microglia cells from a proinflammatory to an anti-inflammatory immunophenotype [75]. In the MPP^+^ rat model, Nam et al. showed that the neuroprotective effect of TRPV1 activation is mediated by ciliary neurotrophic factor (CNTF), released by astrocytes, and its receptor (CNTFR*α*) localized in dopaminergic neurons. Additionally, patients with PD exhibit increased expression of the TRPV1 receptor and CNTF, suggesting the TRPV1 receptor as a therapeutic target for PD [76].

Furthermore, it has been demonstrated that the neuroprotective effect of capsaicin may result from an interaction between the TRPV1 receptor and cannabinoid receptors. This is supported by the observation that the pretreatment with AM251 (CB1 antagonist, 0.1 mg/kg, i.p.) and AM630 (CB2 antagonist, 0.1 mg/kg, i.p.) partially reversed the neuroprotective effect of capsaicin (0.5 mg/kg, i.p.) in mice subjected to acute treatment with MPTP (80 mg/kg, i.p.) [77].

### 4.5. GPR55

The GPR55 receptor is coupled to the activation of the *G*_*α*12/13_ and *G*_*αq*_ proteins, whose activation promotes the release of intracellular calcium (Ca^2+^) from the endoplasmic reticulum and the activation of the ERK pathway. This pathway modulates cellular parameters such as proliferation, differentiation, cell migration, and physiological processes like blood pressure, bone density, inflammation, and energy balance and exhibits antiepileptic action [78]. Due to its abundant expression in the striatum, the GPR55 receptor appears to play a role in the modulation of motor function [79]. Studies conducted by Celorrio et al. demonstrated that GPR55 receptor activation, through agonism with abnormal cannabidiol (a synthetic isomer of cannabidiol with high affinity towards GPR55), prevents dopaminergic cell death, motor deficits, and damage-associated morphological changes in microglia in a chronic MPTPp model in C57BL/6 mice [80]. Additionally, agonism of the GPR55 receptor with VCE-006.1 has been shown to reverse motor deficit induced by 6-OHDA in pole test and cylinder rearing tests, as well as prevent the loss of dopaminergic neurons and inhibit the reactivity of glial cells in SN*pc* [81]. However, the signaling pathways associated with these neuroprotective effects remain unknown.

It is crucial to note that the antiparkinsonian effects of the GPR55 receptor have been controversial. In a study conducted by Fatemi et al. in 2021, using a model of hemiparkinsonism induced by 6-OHDA, intrastriatal administration of GPR55 agonists (LPI, 1 and 5 *μ*g) and antagonists (ML193, 1 and 5 *μ*g) caused an improvement in motor coordination and attenuated sensorimotor deficits in rats. This suggests that GPR55 may have a modulatory role in PD and provides new insights into its involvement in controlling balance, movement, and sensorimotor skills [82].

## 5. Neuroprotection on Clinical Evidence?

The neuroprotective activity of the ECS has not been assessed directly in clinical assays, if we consider a strict, cellular biology-oriented meaning of the term. In part given the technical and ethical complexity of measuring such a feature on humans (it would be necessary to assess dopaminergic viable cell numbers *in vivo* in humans without or with very little adverse effects), there are very few clinical trials that search for this niche definition of neuroprotection [83]. Nevertheless, the consideration of a more broad, operational-oriented definition of neuroprotective activity as the slowing or halt of neurological disease features and symptoms leads us to point out several studies involving diverse drugs targeting the ECS. Regarding this, there are some works that highly emphasize the evidence on the enhancement of ECS signaling, postulating the benefits for targeting it with diverse pharmacological approaches affecting variated targets related to, or that are part of the system itself abovementioned. In Table 2, we summarize their findings along with the components of the ECS presumably affected by the drugs of intervention.

## 6. Conclusions

Considering current evidence, the ECS emerges as a promising therapeutic target for managing PD, primarily owing to its neuroprotective effects, prominently mediated through anti-inflammatory mechanisms. This is particularly significant since neuroinflammation stands out as a hallmark of PD, and extensive preclinical studies have consistently demonstrated that modulating this inflammatory process mitigates the progression of dopaminergic neuronal death. However, the mechanism underlying how the ECS exerts neuroprotection and anti-inflammatory/immunomodulatory actions remains poorly described, highlighting the need for more comprehensive studies on this matter. Concerning the neuroprotective effect of CB1 receptors in parkinsonism models, the evidence is limited, contradictory, and inconclusive, particularly regarding elucidating the involved mechanisms. Further research is needed to address these gaps and provide a clearer understanding of the specific ways in which the ECS contributes to neuroprotection in the context of parkinsonism.

## Figures and Tables

**Table 1:** Differential alterations in diverse components of the endocannabinoid system in Parkinson's disease and parkinsonism models.

Type and component of the ECS		Alteration	Experimental subject/model/sample	References
Ligands	AEA	Augmentation	CSF of patients	[22, 24]
			Monkeys and rats	[66]
			Animal models	[27, 28]
	2-AG	Diminishment	CSF of patients	[23]
		Augmentation	Rat 6-OHDA and reserpine models	[8, 27]
	OEA	Augmentation	Animal models	[27, 28]
	PEA	Augmentation	Animal models	[27]
		Diminishment	Rat 6-OHDA model	[28]
			Mice model	[60]

Production enzymes	NAAA	Augmentation	Rat 6-OHDA model	[60]
			Mice model	
	DAGL	Loss of function	Patients (hereditary Parkinson's)	[20]
		Gene KO	Mice model	

Degradation enzymes	FAAH	Decreased activity	Rats 6-OHDA model	[25]
	MAGL	Augmentation	Brains postmortem	[29]

Receptors	CB1	Diminishment	Blood of patients	[30]
		+/−	Rats 6-OHDA model	[31]
	CB2	Augmentation	Animal models of PD	[28, 61, 62]

**Table 2:** Presuming neuroprotective activity on clinical studies evidenced by improvement on symptoms.

Components of the ECS targeted	Substance(s) employed, dosage, and administering route	Effects observed	References
CB1 and CB2 agonism	Nabilone 0.03 mg oral	Reduction of rush dyskinesia disability, painful dystonia, and time	[84]
Inverse agonist to CB1	Rimonabant 20 mg oral	No improvement of motor symptoms	[85]
All components on ECS affected and many other targets	Whole cannabis smoked	Subjective improvement of rest tremor bradykinesia and dyskinesias	[86]
All components on ECS affected and many other targets	Cannador oral capsule (ethanolic extract containing 2.5 mg of delta-9-tetrahydrocannabinol (THC) and 1.25 mg of cannabidiol (CBD))	No improvement in dyskinesias	[87]
CB1 and CB2 agonism, FAAH inhibition, and PPAR and TRPV modulated activities	CBD 150 mg oral	Reduction of psychotic symptoms	[88]
All components on ECS affected and many other targets	Whole cannabis smoked	Improvement on reducing tremors, rigidity, and bradykinesia, as well as sleep and pain	[89]
CB1 and CB2 agonism, FAAH inhibition, and PPAR- and TRPV-modulated activities	CBD 75 and 300 mg oral	Reduction of disorder related REM sleep behavior	[90]
CB1 and CB2 agonism, FAAH inhibition, and PPAR- and TRPV-modulated activities	CBD 75 and 300 mg oral	No improved motor but improved in quality of life on highest dosage	[91]
All components on ECS affected and many other targets	Cannabis different dosage (patient survey)	Improvement of mood and sleep and of motor symptoms	[25, 92]
CB1 and CB2 agonism	Nabilone 0.25 mg to 1 mg oral	Beneficial effects on sleep disorders associated with PD	[93]
CB1 and CB2 agonism, FAAH inhibition, and PPAR- and TRPV-modulated activities	CBD 300 mg oral	Decrement in anxiety and tremor amplitude on PD patients	[94]
CB1 and CB2 agonism	Nabilone 0.25 mg to 2 mg oral	Beneficial effects on sleep outcomes in PD patients	[95]

## Data Availability

Any information regarding the contents of this paper will be properly disclaimed by the correspondence author upon reasonable request.

## References

[1] Sveinbjornsdottir S. (2016). The clinical symptoms of Parkinson’s disease. *Journal of Neurochemistry*.

[2] Nutt J. G., Wooten G. F. (2005). Diagnosis and initial management of Parkinson’s disease. *New England Journal of Medicine*.

[3] Vasincu A., Rusu R. N., Ababei D. C. (2022). Endocannabinoid modulation in neurodegenerative diseases: in pursuit of certainty. *Biology*.

[4] Armstrong M. J., Okun M. S. (2020). Diagnosis and treatment of Parkinson disease: a review. *JAMA*.

[5] Wang M., Liu H., Ma Z. (2022). Roles of the cannabinoid system in the basal ganglia in Parkinson’s disease. *Frontiers in Cellular Neuroscience*.

[6] Melis M., Pillolla G., Bisogno T. (2006). Protective activation of the endocannabinoid system during ischemia in dopamine neurons. *Neurobiology of Disease*.

[7] Cristino L., Bisogno T., Di Marzo V. (2020). Cannabinoids and the expanded endocannabinoid system in neurological disorders. *Nature Reviews Neurology*.

[8] Di Marzo V., Hill M. P., Bisogno T., Crossman A. R., Brotchie J. M. (2000). Enhanced levels of endogenous cannabinoids in the globus pallidus are associated with a reduction in movement in an animal model of Parkinson’s disease. *The FASEB Journal*.

[9] Hohmann A. G., Suplita R. L. (2006). Endocannabinoid mechanisms of pain modulation. *The AAPS Journal*.

[10] Sagar D. R., Gaw A. G., Okine B. N. (2009). Dynamic regulation of the endocannabinoid system: implications for analgesia. *Molecular Pain*.

[11] Guindon J., Hohmann A. G. (2009). The endocannabinoid system and pain. *CNS and Neurological Disorders-Drug Targets*.

[12] Battista N., Di Tommaso M., Bari M., Maccarrone M. (2012). The endocannabinoid system: an overview. *Frontiers in Behavioral Neuroscience*.

[13] Jarvis S., Rassmussen S., Winters B. (2017). Role of the endocannabinoid system and medical cannabis. *The Journal for Nurse Practitioners*.

[14] Muller C., Morales P., Reggio P. H. (2018). Cannabinoid ligands targeting TRP channels. *Frontiers in Molecular Neuroscience*.

[15] Burgaz S., Garcia C., Gomez-Canas M., Munoz E., Fernandez-Ruiz J. (2019). Development of an oral treatment with the PPAR-gamma-acting cannabinoid VCE-003.2 against the inflammation-driven neuronal deterioration in experimental Parkinson’s disease. *Molecules*.

[16] De Petrocellis L., Di Marzo V. (2009). An introduction to the endocannabinoid system: from the early to the latest concepts. *Best Practice and Research Clinical Endocrinology and Metabolism*.

[17] Muccioli G. G. (2010). Endocannabinoid biosynthesis and inactivation, from simple to complex. *Drug Discovery Today*.

[18] El Manira A., Kyriakatos A. (2010). The role of endocannabinoid signaling in motor control. *Physiology*.

[19] Zogopoulos P., Vasileiou I., Patsouris E., Theocharis S. E. (2013). The role of endocannabinoids in pain modulation. *Fundamental and Clinical Pharmacology*.

[20] Liu Z., Yang N., Dong J. (2022). Deficiency in endocannabinoid synthase DAGLB contributes to early onset Parkinsonism and murine nigral dopaminergic neuron dysfunction. *Nature Communications*.

[21] Zou S., Kumar U. (2018). Cannabinoid receptors and the endocannabinoid system: signaling and function in the central nervous system. *International Journal of Molecular Sciences*.

[22] Pisani V., Madeo G., Tassone A. (2011). Homeostatic changes of the endocannabinoid system in Parkinson’s disease. *Movement Disorders*.

[23] Marchioni C., Santos-Lobato B. L., Queiroz M. E. C., Crippa J. A. S., Tumas V. (2020). Endocannabinoid levels in patients with Parkinson’s disease with and without levodopa-induced dyskinesias. *Journal of Neural Transmission*.

[24] van der Stelt M., Di Marzo V. (2005). Cannabinoid receptors and their role in neuroprotection. *Neuro Molecular Medicine*.

[25] Gubellini P., Picconi B., Bari M. (2002). Experimental parkinsonism alters endocannabinoid degradation: implications for striatal glutamatergic transmission. *Journal of Neuroscience*.

[26] Maccarrone M., Gubellini P., Bari M. (2003). Levodopa treatment reverses endocannabinoid system abnormalities in experimental parkinsonism. *Journal of Neurochemistry*.

[27] Concannon R. M., Okine B. N., Finn D. P., Dowd E. (2015). Differential upregulation of the cannabinoid CB(2) receptor in neurotoxic and inflammation-driven rat models of Parkinson’s disease. *Experimental Neurology*.

[28] Concannon R. M., Okine B. N., Finn D. P., Dowd E. (2016). Upregulation of the cannabinoid CB2 receptor in environmental and viral inflammation-driven rat models of Parkinson’s disease. *Experimental Neurology*.

[29] Navarrete F., Garcia-Gutierrez M. S., Aracil-Fernandez A., Lanciego J. L., Manzanares J. (2018). Cannabinoid CB1 and CB2 receptors, and monoacylglycerol lipase gene expression alterations in the basal ganglia of patients with Parkinson’s disease. *Neurotherapeutics*.

[30] Van Laere K., Casteels C., Lunskens S. (2012). Regional changes in type 1 cannabinoid receptor availability in Parkinson’s disease in vivo. *Neurobiology of Aging*.

[31] Walsh S., Mnich K., Mackie K., Gorman A. M., Finn D. P., Dowd E. (2010). Loss of cannabinoid CB1 receptor expression in the 6-hydroxydopamine-induced nigrostriatal terminal lesion model of Parkinson’s disease in the rat. *Brain Research Bulletin*.

[32] Braak H., Sastre M., Del Tredici K. (2007). Development of *α*-synuclein immunoreactive astrocytes in the forebrain parallels stages of intraneuronal pathology in sporadic Parkinson’s disease. *Acta Neuropathologica*.

[33] Lee H. J., Suk J. E., Patrick C. (2010). Direct transfer of *α*-synuclein from neuron to astroglia causes inflammatory responses in synucleinopathies. *Journal of Biological Chemistry*.

[34] Gu X. L., Long C. X., Sun L., Xie C., Lin X., Cai H. (2010). Astrocytic expression of Parkinson’s disease-related A53T *α*-synuclein causes neurodegeneration in mice. *Molecular Brain*.

[35] Long-Smith C. M., Sullivan A. M., Nolan Y. M. (2009). The influence of microglia on the pathogenesis of Parkinson’s disease. *Progress in Neurobiology*.

[36] Croisier E., Moran L. B., Dexter D. T., Pearce R. K., Graeber M. B. (2005). Microglial inflammation in the parkinsonian substantia nigra: relationship to alpha-synuclein deposition. *Journal of Neuroinflammation*.

[37] Fernandez-Ruiz J., Pazos M. R., Garcia-Arencibia M., Sagredo O., Ramos J. A. (2008). Role of CB2 receptors in neuroprotective effects of cannabinoids. *Molecular and Cellular Endocrinology*.

[38] Mounsey R. B., Mustafa S., Robinson L. (2015). Increasing levels of the endocannabinoid 2-AG is neuroprotective in the 1-methyl-4-phenyl-1,2,3,6-tetrahydropyridine mouse model of Parkinson’s disease. *Experimental Neurology*.

[39] Kreitzer A. C., Malenka R. C. (2007). Endocannabinoid-mediated rescue of striatal LTD and motor deficits in Parkinson’s disease models. *Nature*.

[40] Celorrio M., Fernandez-Suarez D., Rojo-Bustamante E. (2016). Fatty acid amide hydrolase inhibition for the symptomatic relief of Parkinson’s disease. *Brain, Behavior, and Immunity*.

[41] Pasquarelli N., Porazik C., Bayer H. (2017). Contrasting effects of selective MAGL and FAAH inhibition on dopamine depletion and GDNF expression in a chronic MPTP mouse model of Parkinson’s disease. *Neurochemistry International*.

[42] Nomura D. K., Morrison B. E., Blankman J. L. (2011). Endocannabinoid hydrolysis generates brain prostaglandins that promote neuroinflammation. *Science*.

[43] Fernandez-Suarez D., Celorrio M., Riezu-Boj J. I. (2014). The monoacylglycerol lipase inhibitor JZL184 is neuroprotective and alters glial cell phenotype in the chronic MPTP mouse model. *Neurobiology of Aging*.

[44] Escamilla-Ramirez A., Garcia E., Palencia-Hernandez G. (2017). URB597 and the cannabinoid WIN55,212-2 reduce behavioral and neurochemical deficits induced by MPTP in mice: possible role of redox modulation and NMDA receptors. *Neurotoxicity Research*.

[45] Chang J. W., Niphakis M. J., Lum K. M. (2012). Highly selective inhibitors of monoacylglycerol lipase bearing a reactive group that is bioisosteric with endocannabinoid substrates. *Chemistry and Biology*.

[46] Aymerich M. S., Rojo-Bustamante E., Molina C., Celorrio M., Sanchez-Arias J. A., Franco R. (2016). Neuroprotective effect of JZL184 in MPP(+)-Treated SH-SY5Y cells through CB2 receptors. *Molecular Neurobiology*.

[47] Viveros-Paredes J. M., Gonzalez-Castaneda R. E., Escalante-Castaneda A., Tejeda-Martinez A. R., Castaneda-Achutigui F., Flores-Soto M. E. (2019). Effect of inhibition of fatty acid amide hydrolase on MPTP-induced dopaminergic neuronal damage. *Neurologia*.

[48] Dionisi M., Alexander S. P., Bennett A. J. (2012). Oleamide activates peroxisome proliferator-activated receptor gamma (PPAR*γ*) in vitro. *Lipids in Health and Disease*.

[49] Kainu T., Wikstrom A. C., Gustafsson J. A., Pelto-Huikko M. (1994). Localization of the peroxisome proliferator-activated receptor in the brain. *Neuro Report*.

[50] Cullingford T. E., Bhakoo K., Peuchen S., Dolphin C. T., Patel R., Clark J. B. (1998). Distribution of mRNAs encoding the peroxisome proliferator‐activated receptor *α*, *β*, and *γ* and the retinoid X receptor *α*, *β*, and *γ* in rat central nervous system. *Journal of Neurochemistry*.

[51] Moreno S., Farioli-Vecchioli S., Ceru M. P. (2004). Immunolocalization of peroxisome proliferator-activated receptors and retinoid X receptors in the adult rat CNS. *Neuroscience*.

[52] Galan-Rodriguez B., Suarez J., Gonzalez-Aparicio R. (2009). Oleoylethanolamide exerts partial and dose-dependent neuroprotection of substantia nigra dopamine neurons. *Neuropharmacology*.

[53] Bordet R., Ouk T., Petrault O. (2006). PPAR: a new pharmacological target for neuroprotection in stroke and neurodegenerative diseases. *Biochemical Society Transactions*.

[54] Schmidt A., Vogel R., Holloway M. K. (1999). Transcription control and neuronal differentiation by agents that activate the LXR nuclear receptor family. *Molecular and Cellular Endocrinology*.

[55] Delerive P., Fruchart J. C., Staels B. (2001). Peroxisome proliferator-activated receptors in inflammation control. *Journal of Endocrinology*.

[56] Lleo A., Galea E., Sastre M. (2007). Molecular targets of non-steroidal anti-inflammatory drugs in neurodegenerative diseases. *Cellular and Molecular Life Sciences*.

[57] Esposito E., Impellizzeri D., Mazzon E., Paterniti I., Cuzzocrea S. (2012). Neuroprotective activities of palmitoylethanolamide in an animal model of Parkinson’s disease. *PLoS One*.

[58] Crupi R., Impellizzeri D., Cordaro M. (2018). N-Palmitoylethanolamide prevents parkinsonian phenotypes in aged mice. *Molecular Neurobiology*.

[59] Avagliano C., Russo R., De Caro C. (2016). Palmitoylethanolamide protects mice against 6-OHDA-induced neurotoxicity and endoplasmic reticulum stress: in vivo and in vitro evidence. *Pharmacological Research*.

[60] Palese F., Pontis S., Realini N. (2022). Targeting NAAA counters dopamine neuron loss and symptom progression in mouse models of parkinsonism. *Pharmacological Research*.

[61] Basile M. S., Mazzon E. (2022). The role of cannabinoid type 2 receptors in Parkinson’s disease. *Biomedicines*.

[62] Viveros-Paredes J. M., Gonzalez-Castaneda R. E., Gertsch J. (2017). Neuroprotective effects of *β*-caryophyllene against dopaminergic neuron injury in a murine model of Parkinson’s disease induced by MPTP. *Pharmaceuticals*.

[63] Price D. A., Martinez A. A., Seillier A. (2009). WIN55,212-2, a cannabinoid receptor agonist, protects against nigrostriatal cell loss in the 1-methyl-4-phenyl-1,2,3,6-tetrahydropyridine mouse model of Parkinson’s disease. *European Journal of Neuroscience*.

[64] Yu H., Liu X., Chen B. (2021). The neuroprotective effects of the CB2 agonist GW842166x in the 6-OHDA mouse model of Parkinson’s disease. *Cells*.

[65] Wang M., Liu M., Ma Z. (2023). Cannabinoid type 2 receptor activation inhibits MPP(+)-induced M1 differentiation of microglia through activating PI3K/Akt/Nrf2 signal pathway. *Molecular Biology Reports*.

[66] Maccarrone M., Battista N., Centonze D. (2007). The endocannabinoid pathway in Huntington’s disease: a comparison with other neurodegenerative diseases. *Progress in Neurobiology*.

[67] Jamwal S., Blackburn J. K., Elsworth J. D. (2021). PPAR*γ*/PGC1*α* signaling as a potential therapeutic target for mitochondrial biogenesis in neurodegenerative disorders. *Pharmacology and Therapeutics*.

[68] Warden A., Truitt J., Merriman M. (2016). Localization of PPAR isotypes in the adult mouse and human brain. *Scientific Reports*.

[69] Zamanian M. Y., Terefe E. M., Taheri N. (2023). Neuroprotective and anti-inflammatory effects of pioglitazone on Parkinson’s disease: a comprehensive narrative review of clinical and experimental findings. *CNS and Neurological Disorders: Drug Targets*.

[70] Blackburn J. K., Jamwal S., Wang W., Elsworth J. D. (2022). Pioglitazone transiently stimulates paraoxonase-2 expression in male nonhuman primate brain: implications for sex-specific therapeutics in neurodegenerative disorders. *Neurochemistry International*.

[71] Burgaz S., Garcia C., Gomez-Canas M., Rolland A., Munoz E., Fernandez-Ruiz J. (2021). Neuroprotection with the cannabidiol quinone derivative VCE-004.8 (EHP-101) against 6-hydroxydopamine in cell and murine models of Parkinson’s disease. *Molecules*.

[72] Burgaz S., Garcia C., Gomez-Canas M. (2021). Neuroprotection with the cannabigerol quinone derivative VCE-003.2 and its analogs CBGA-Q and CBGA-Q-Salt in Parkinson’s disease using 6-hydroxydopamine-lesioned mice. *Molecular and Cellular Neuroscience*.

[73] Garcia C., Gomez-Canas M., Burgaz S. (2018). Benefits of VCE-003.2, a cannabigerol quinone derivative, against inflammation-driven neuronal deterioration in experimental Parkinson’s disease: possible involvement of different binding sites at the PPAR*γ* receptor. *Journal of Neuroinflammation*.

[74] Toth A., Blumberg P. M., Boczan J. (2009). Anandamide and the vanilloid receptor (TRPV1). *Vitamins and Hormones*.

[75] Park E. S., Kim S. R., Jin B. K. (2012). Transient receptor potential vanilloid subtype 1 contributes to mesencephalic dopaminergic neuronal survival by inhibiting microglia-originated oxidative stress. *Brain Research Bulletin*.

[76] Nam J. H., Park E. S., Won S. Y. (2015). TRPV1 on astrocytes rescues nigral dopamine neurons in Parkinson’s disease via CNTF. *Brain*.

[77] Wi R., Chung Y. C., Jin B. K., Duan L. (2020). Functional crosstalk between CB and TRPV1 receptors protects nigrostriatal dopaminergic neurons in the MPTP model of Parkinson’s disease. *Journal of Immunology Research*.

[78] Marichal-Cancino B. A., Fajardo-Valdez A., Ruiz-Contreras A. E., Mendez-Diaz M., Prospero-Garcia O. (2017). Advances in the physiology of GPR55 in the central nervous system. *Current Neuropharmacology*.

[79] Wu C. S., Chen H., Sun H. (2013). GPR55, a G-protein coupled receptor for lysophosphatidylinositol, plays a role in motor coordination. *PLoS One*.

[80] Celorrio M., Rojo-Bustamante E., Fernandez-Suarez D. (2017). GPR55: a therapeutic target for Parkinson’s disease?. *Neuropharmacology*.

[81] Burgaz S., Garcia C., Gonzalo-Consuegra C. (2021). Preclinical investigation in neuroprotective effects of the GPR55 ligand VCE-006.1 in experimental models of Parkinson’s disease and amyotrophic lateral sclerosis. *Molecules*.

[82] Fatemi I., Abdollahi A., Shamsizadeh A., Allahtavakoli M., Roohbakhsh A. (2021). The effect of intra-striatal administration of GPR55 agonist (LPI) and antagonist (ML193) on sensorimotor and motor functions in a Parkinson’s disease rat model. *Acta Neuropsychiatrica*.

[83] Ravina B. M., Fagan S. C., Hart R. G. (2003). Neuroprotective agents for clinical trials in Parkinson’s disease: a systematic assessment. *Neurology*.

[84] Sieradzan K. A., Fox S. H., Hill M., Dick J. P., Crossman A. R., Brotchie J. M. (2001). Cannabinoids reduce levodopa-induced dyskinesia in Parkinson’s disease: a pilot study. *Neurology*.

[85] Mesnage V., Houeto J. L., Bonnet A. M. (2004). Neurokinin B, neurotensin, and cannabinoid receptor antagonists and Parkinson disease. *Clinical Neuropharmacology*.

[86] Venderova K., Ruzicka E., Vorisek V., Visnovsky P. (2004). Survey on cannabis use in Parkinson’s disease: subjective improvement of motor symptoms. *Movement Disorders*.

[87] McSherry J. W. (2005). Cannabis for dyskinesia in Parkinson disease: a randomized double-blind crossover study. *Neurology*.

[88] Zuardi A. W., Crippa J. A., Hallak J. E. (2009). Cannabidiol for the treatment of psychosis in Parkinson’s disease. *Journal of Psychopharmacology*.

[89] Lotan I., Treves T. A., Roditi Y., Djaldetti R. (2014). Cannabis (medical marijuana) treatment for motor and non-motor symptoms of Parkinson disease: an open-label observational study. *Clinical Neuropharmacology*.

[90] Chagas M. H., Eckeli A. L., Zuardi A. W. (2014). Cannabidiol can improve complex sleep-related behaviours associated with rapid eye movement sleep behaviour disorder in Parkinson’s disease patients: a case series. *Journal of Clinical Pharmacy and Therapeutics*.

[91] Chagas M. H., Zuardi A. W., Tumas V. (2014). Effects of cannabidiol in the treatment of patients with Parkinson’s disease: an exploratory double-blind trial. *Journal of Psychopharmacology*.

[92] Finseth T. A., Hedeman J. L., Brown R. P., Johnson K. I., Binder M. S., Kluger B. M. (2015). Self-reported efficacy of cannabis and other complementary medicine modalities by Parkinson’s disease patients in Colorado. *Evidence-Based Complementary and Alternative Medicine*.

[93] Peball M., Werkmann M., Ellmerer P. (2019). Nabilone for non-motor symptoms of Parkinson’s disease: a randomized placebo-controlled, double-blind, parallel-group, enriched enrolment randomized withdrawal study (The NMS-Nab Study). *Journal of Neural Transmission*.

[94] de Faria S. M., de Morais Fabricio D., Tumas V. (2020). Effects of acute cannabidiol administration on anxiety and tremors induced by a Simulated Public Speaking Test in patients with Parkinson’s disease. *Journal of Psychopharmacology*.

[95] Peball M., Seppi K., Krismer F. (2022). Effects of nabilone on sleep outcomes in patients with Parkinson’s disease: a post-hoc analysis of NMS-nab study. *Movement Disorders Clinical Practice*.

